# High Serum miR-19a Levels Are Associated with Inflammatory Breast Cancer and Are Predictive of Favorable Clinical Outcome in Patients with Metastatic HER2^+^ Inflammatory Breast Cancer

**DOI:** 10.1371/journal.pone.0083113

**Published:** 2014-01-08

**Authors:** Simone Anfossi, Antonio Giordano, Hui Gao, Evan N. Cohen, Sanda Tin, Qiong Wu, Raul J. Garza, Bisrat G. Debeb, Ricardo H. Alvarez, Vicente Valero, Gabriel N. Hortobagyi, George A. Calin, Naoto T. Ueno, Wendy A. Woodward, James M. Reuben

**Affiliations:** 1 Department of Hematopathology, The University of Texas MD Anderson Cancer Center, Houston, Texas, United States of America; 2 Morgan Welch Inflammatory Breast Cancer Research Program and Clinic, The University of Texas MD Anderson Cancer Center, Houston, Texas, United States of America; 3 Department of Radiation Oncology, The University of Texas MD Anderson Cancer Center, Houston, Texas, United States of America; 4 Department of Breast Medical Oncology, The University of Texas MD Anderson Cancer Center, Houston, Texas, United States of America; 5 Department of Experimental Therapeutics, The University of Texas MD Anderson Cancer Center, Houston, Texas, United States of America; 6 The University of Texas Graduate School of Biomedical Sciences at Houston, The University of Texas MD Anderson Cancer Center, Houston, Texas, United States of America; Federico II University of Naples, Italy, Italy

## Abstract

**Introduction:**

Altered serum microRNA (miRNA) levels may be correlated with a dysregulated expression pattern in parental tumor tissue and reflect the clinical evolution of disease. The overexpression of miR-21, miR-10b, and miR-19a is associated with the acquisition of malignant characteristics (increased tumor cell proliferation, migration, invasion, dissemination, and metastasis); thus, we determined their utility as serum biomarkers for aggressive breast cancer (HER2-overexpressed or -amplified [HER2^+^] and inflammatory breast cancer [IBC]).

**Experimental Design:**

In this prospective study, we measured miR-21, miR-10b, and miR-19a levels using quantitative reverse transcriptase-polymerase chain reaction in the serum of 113 breast cancer patients and determined their association with clinicopathologic factors and clinical outcome. Thirty healthy donors with no history of cancer were enrolled as controls.

**Results:**

Patients with non-metastatic HER2^+^ breast cancer had higher serum miR-21 median levels than patients with non-metastatic HER2^−^ disease (p = 0.044); whereas patients with metastatic HER2^+^ breast cancer had higher serum miR-10b median levels than patients with metastatic HER2^−^ disease (p = 0.0004). There were no significant differences in serum miR-19a median levels between HER2^+^ and HER2^−^ groups, regardless of the presence of metastases. High serum miR-19a levels were associated with IBC (p = 0.039). Patients with metastatic IBC had significantly higher serum miR-19a median levels than patients with metastatic non-IBC (p = 0.019). Finally, high serum miR-19a levels were associated with longer progression-free survival time (10.3 vs. 3.2 months; p = 0.022) and longer overall survival time (median not reached vs. 11.2 months; p = 0.003) in patients with metastatic HER2^+^ IBC.

**Conclusion:**

High levels of miR-21 and miR-10b were present in the serum of patients with non-metastatic and metastatic HER2^+^ breast cancer, respectively. High levels of serum miR-19a may represent a biomarker for IBC that is predictive for favorable clinical outcome in patients with metastatic HER2^+^ IBC.

## Introduction

Despite improvements in screening, more effective and less toxic treatments, and a decreasing disease incidence, breast cancer still remains the second leading cause of death among women in the United States [1]. Inflammatory breast cancer (IBC) is a rare, phenotypically distinct, highly aggressive form of locally advanced breast cancer that comprises approximately 5% of all breast cancer cases [2]; according to the National Cancer Institute's Surveillance, Epidemiology, and End Results (SEER), its incidence is increasing. IBC is characterized by high invasive and angiogenic ability, fast progression, and high propensity to disseminate in the dermal lymphatics and metastasize to distant organs [3]. These features confer to IBC an extremely high metastatic potential, that is responsible for its worse prognosis, with a 5-year overall survival rate of only 40.5% compared with 85% in stage III non-IBC patients [4]. To date, no unique molecular diagnostic or prognostic biomarkers have been identified for IBC.

HER2-overexpression or amplification (HER2^+^) is found in approximately 30% of breast cancers and is associated with increased tumor aggressive behavior and poor outcome. Although anti-HER2 treatment with trastuzumab can prolong HER2^+^ breast cancer patients' survival [5], most of the patients, who initially respond, develop resistance to trastuzumab within one year of the beginning of the treatment [6]. Because drug resistance and metastasis remain the major causes of death in cancer patients, identifying and characterizing patients at risk for resistance is essential to establishing more effective and personalized treatments. The development of highly sensitive, specific, minimally invasive tools may help improve diagnosis and monitor and predict treatment response. Therefore, new diagnostic and prognostic biomarkers are needed.

MiRNAs are a recently discovered class of small non-coding RNA molecules (typically 18–24 nucleotides in size) that play a role in regulating important cell processes, such as proliferation, apoptosis, migration, and differentiation. More than 50% of miRNAs are located in chromosomal regions that are subject to genetic alterations in human cancers, such as deletion, amplification, translocation, and mutation [7]. Hence, tumor cells may undergo genetic changes that lead to an aberrant expression pattern compared with normal tissues [8]. An altered miRNA pattern is also observed in the serum of patients with various cancers, including B-cell lymphoma [9], prostate cancer [10], colorectal cancer [11], lung cancer [12], ovarian cancer [13], and breast cancer [14]. MiRNAs can be released passively by tumor cell lysis/apoptosis or actively by live cell secretion [15]–[17]. Hence, aberrant levels of miRNA in the blood of cancer patients may reflect pathological changes associated with disease development and may be correlated with the dysregulated pattern of the primary or metastatic parental tumor [18]. Serum miRNAs are present in the peripheral blood in two highly stable forms of circulating cell-free nucleic acids: 1) encapsulated in membrane-bound vesicles (exosomes and microvesicles) [19]; and 2) associated with Argonaute2 protein [20]. These characteristics make serum miRNAs highly resistant to harsh conditions, such as low and high pH, boiling temperatures, freeze-thaw cycles, and RNase digestion [10], [21]. Collectively, these characteristics suggest that circulating miRNAs are suitable biomarkers for diagnosing and monitoring cancer.

MiR-21, miR-10b, and miR-19a are overexpressed in breast cancer and play an important role in tumor progression and metastasis development. In particular, miR-21 regulates tumor cell growth, proliferation, migration, apoptosis [8], [22], [23], and angiogenesis by targeting phosphatase and tensin homolog (PTEN) and the phosphoinositide 3-kinase/AKT pathway [24]. MiR-21 overexpression is also associated with advanced clinical stage [25] and trastuzumab resistance by targeting PTEN [26]. MiR-10b is highly expressed in metastatic human breast cancer cells; it regulates migration and invasion and initiates distant metastasis [27]. MiR-19a was found to be the main oncogenic component of the miR-17-92 cluster by downregulating the tumor suppressor PTEN [28], [29]; it was overexpressed in a mouse model of human breast cancer bone metastasis [30] and induced enhanced neoangiogenesis by targeting the anti-angiogenic regulator thrombospondin-1 (Tsp-1) [31].

To date, there are no valuable serum biomarkers able to distinguish IBC from non-IBC and predict clinical outcome of patients with IBC and HER2^+^ breast cancer. As miR-21, miR-10b, and miR-19a regulate metastasis formation, angiogenesis, invasion and these clinicopathologic characteristics are enhanced in IBC, we hypothesized that serum miR-21, miR-10b, and miR-19a levels are increased in patients with IBC. Moreover, as the loss of PTEN is involved in the development of resistance to anti-HER2 treatment and PTEN is a target of miR-21 and miR-19a, we hypothesized that high levels of these two miRNAs can be associated with poor clinical outcome.

In this study, we determined whether the levels of miR-21, miR-10b, and miR-19a in the serum of breast cancer patients would be useful as diagnostic and prognostic biomarkers for patients with IBC and HER2^+^ breast cancer.

## Patients and Methods

### Ethics statement

The study has been approved by the Institutional Review Board (IRB) at The University of Texas MD Anderson Cancer Center, and adhered to the tenets of the Declaration of Helsinki. Written informed consent was obtained from each participant prior to sample collection.

### Patients' characteristics

In this prospective study, 113 breast cancer patients were recruited from October 2008 to May 2010 in two laboratory-based protocols approved by MD Anderson Cancer Center, Houston, TX. Patients with newly diagnosed IBC stage III; IBC stage IV; non-IBC stage II, III, IV; and HER2^+^ breast cancer were considered eligible. Serum was collected from all patients at the beginning of the study (before a new line of therapy for patients with metastatic disease [M1] and before first-line therapy for patients with non-metastatic disease [M0]). HER2 status was evaluated by immunohistochemical (IHC) or by fluorescence in situ hybridization (FISH) assays. HER2 overexpression or amplification (HER2^+^) was defined as an IHC staining score of 3+ (i.e., strong membranous staining in at least 10% of cells) or as gene amplification found on FISH analysis, as previously reported [32]. A gene copy-to-chromosome 17 centromere (CEP)-17 ratio of >2.0 was considered amplified. Patients' clinical and histopathological information is summarized in Table 1. Serum samples were also collected from 30 age-matched healthy donors (HDs) recruited at MD Anderson Cancer Center. These samples were included as a control group for the miRNA analyses. The HDs were explained the purpose of the study and agreed to sign a written informed consent approved by IRB stating that they did not have history of cancer prior the blood collection. As a monitor of general health status, HDs provided a sample of blood for hematological profile including a complete blood count (CBC) with leukocyte differential analysis that was performed in the CLIA-certified clinical pathology laboratory at MD Anderson Cancer Center.

**Table 1:** Clinical characteristics of breast cancer patients at the beginning of the study and their association with serum miR-21, miR-10b, and miR-19a levels.

		miR-21, n (%)			miR-10b, n (%)			miR-19a, n (%)		
Characteristic	n	Low	High	*P*	Low	High	*P*	Low	High	*P*
Age (53 years)	113									
<45	24	11 (18)	13 (25)	.489	18 (21)	6 (21)	1.000	7 (13)	17 (28)	.065
≥45	89	50 (82)	39 (75)		67 (79)	22 (79)		46 (87)	43 (72)	
Race										
Asian	5	3 (5)	2 (4)	.947	5 (6)	0	.566	4 (7)	1 (2)	.210
African-American	6	4 (7)	2 (4)		5 (6)	1 (4)		4 (7)	2 (3)	
Hispanic	10	5 (8)	5 (9)		8 (9)	2 (7)		6 (12)	4 (7)	
Non-Hispanic white	92	49 (80)	43 (83)		67 (79)	25 (89)		39 (74)	53 (88)	
IBC										
Yes	63	32 (52)	31 (62)	.455	49 (58)	14 (53)	.829	24 (45)	39 (65)	**.039**
No	50	29(48)	21 (38)		37 (43)	13 (47)		29(55)	21 (35)	
Stage										
M0	35	22 (36)	13 (25)	.227	31 (36)	4 (14)	**.034**	17 (32)	18 (30)	.841
M1	78	39 (64)	39 (75)		54 (64)	24 (86)		36 (68)	42 (70)	
Grade										
1	3	2 (3)	1 (2)	.867	3 (4)	0	.444	2 (4)	1 (2)	.582
2	29	14 (25)	15 (29)		23 (28)	6 (21)		15 (29)	14 (24)	
3	78	42 (72)	36 (69)		56 (68)	22 (79)		34 (67)	44 (74)	
N/A	3									
Hormone receptor										
Positive	68	40 (66)	28 (54)	.249	55 (65)	13 (46)	.119	36 (68)	32 (53)	.127
Negative	45	21 (34)	24 (46)		30 (35)	15 (54)		17 (32)	28 (47)	
HER2										
Positive	65	31 (51)	34 (65)	.131	40 (47)	25 (89)	**.001**	31 (58)	34 (57)	.851
Negative	48	30 (49)	18 (35)		45 (53)	3 (11)		22 (42)	26 (43)	
Triple negative status										
Yes	18	10 (16)	8 (15)	.100	16 (19)	2 (7)	.233	4 (8)	14 (23)	**.037**
No	95	51 (84)	44 (85)		69 (81)	26 (93)		49 (92)	46 (77)	

### Blood collection and RNA extraction

Ten mL of peripheral blood from breast cancer patients and HDs were collected in BD Vacutainer serum tubes (Becton Dickinson Vacutainer, Franklin Lakes, NJ) and left to clot at ambient temperature for 30 minutes. The serum was separated by centrifugation within 3–4 h after phlebotomy and stored at −80°C in 1 mL aliquots until RNA could be extracted and purified. Total RNA was isolated using the Total RNA Purification Kit (Norgen Biotek Corporation, Thorold, ON, Canada) following the manufacturer's instructions, starting with 100 µL of serum and 1×10^6^ of breast cancer cell lines (see below). The concentration of total RNA in each sample was measured using a NanoDrop 2000 spectrophotometer (Thermo Scientific, Wilmington, DE). The RNA was immediately stored at −80°C.

### Breast cancer cell lines

The human breast cancer cell lines MCF-7 (estrogen receptor-positive [ER^+^], metastatic pleural effusion), SKBR-3 (HER2^+^, metastatic pleural effusion), MDA-231 (ER^−^, progesterone receptor [PR^−^], HER2^−^: triple receptor negative [TN], metastatic pleural effusion), KPL-4 (HER2^+^, metastatic pleural effusion, IBC) were obtained from the American Type Culture Collection (Manassas, VA) and maintained in culture with DMEM/F-12 medium supplemented with 10% fetal bovine serum (Tissue Culture Biologicals, Seal Beach, CA) and 1% of antibiotic-antimycotic 100X (Gibco, Carlsbad, CA). The TN human IBC cell line SUM-149 was kindly provided by co-author, Dr. Naoto T. Ueno (The Morgan Welch Inflammatory Breast Cancer Research Program and Clinic, The University of Texas MD Anderson Cancer Center, Houston, TX), who purchased the cell line from Asterand Inc. (Detroit, MI). SUM-149 cells were maintained in culture with Ham's/F-12 medium, supplemented with 10% fetal bovine serum (Tissue Culture Biologicals), 5 µg/mL insulin, 1 µg/mL hydrocortisone, and 1% of antibiotic-antimycotic 100X (Gibco).

### Conversion of total RNA into cDNA

RNA isolated from serum samples and cell lines was reverse-transcribed to cDNA using the TaqMan MicroRNA Reverse Transcription kit (Applied Biosystems, Foster City, CA) according to the manufacturer's instructions. In brief, 10 ng of total RNA were reverse-transcribed in a total volume reaction of 15 µL containing 1 nM dNTPs, 3.3 U/µL MultiScribe reverse transcriptase, 1× reverse transcription buffer, 0.25 U/µl RNase inhibitor, 1× specific miRNA primer (TaqMan MicroRNA Assays, Applied Biosystems), and nuclease-free water. The reaction was performed using the Veriti Thermal Cycler (Applied Biosystems) at 16°C for 30 minutes, 42°C for 30 minutes, and 85°C for 5 minutes.

### Quantification of miR-192, miR-21, miR-19a, and miR-10b by quantitative reverse transcription-polymerase chain reaction

MiRNA levels were measured by quantitative reverse transcription-polymerase chain reaction (qRT-PCR) using TaqMan MicroRNA assays (Applied Biosystems) according to the manufacturer's instructions. In brief, cDNA was diluted 1∶15 in RNase-free water and added to a final qRT-PCR reaction volume of 10 µL, which contained TaqMan MicroRNA assay primers for each miRNA, TaqMan universal PCR Master Mix No AmpErase UNG, and nuclease-free water. The reaction was performed using 7900HT fast real-time PCR systems (Applied Biosystems) at 95°C for 10 minutes and 40 cycles at 95°C for 15 seconds and 60°C for 60 seconds. After validating miR-192 as reference miRNA, the relative levels of miR-21, miR-19a, and miR-10b was calculated using the equation 2^−ΔCt^, where ΔCt  =  mean Ct_miRNA_ – mean Ct_miR-192_, and Ct  =  threshold cycle. To normalize miR-19a expression in breast cancer cell lines, we used U6 snRNA and calculated the relative expression using the equation 2^−ΔCt^, where ΔCt  =  mean Ct_miR-19a_ – mean Ct_U6snRNA_. To compare the difference between the two reference miRNAs miR-192 and miR-16 in the qRT-PCR normalization of serum miRNAs, the equation 2^−ΔΔCt^ was used to calculate the fold difference of relative serum miRNA levels between breast cancer patients and HDs, where ΔΔCt  =  (mean Ct_miRNA_ – mean Ct_Reference miRNA_)_patients_ – (mean Ct_miRNA_ – mean Ct_ Reference miRNA_)_HDs_.

### Statistical analysis

The differences in miRNA levels and the receiver operating characteristic (ROC) curves were evaluated using GraphPad Prism 5.04 software (GraphPad Software, Inc., San Diego, CA). The non-parametric 2-tailed Mann Whitney-U test was used to perform a statistical analysis of serum miRNA levels, and the 2-tailed Student's *t-*test was used to compare miR-19a expression in breast cancer cell lines. Wilcoxon matched-pairs signed rank test was used to compare the difference between the two reference miRNAs (miR-192 and miR-16) in the normalization of serum miRNA levels calculated by using the equation 2^−ΔΔCt^. ROC curve analyses were used to establish the diagnostic power of serum miRNAs, and the areas under the curves (AUC) were calculated. The Fisher's exact test was used to evaluate the association between serum miRNA levels and clinicopathologic variables. The Kaplan-Meier method was used to evaluate the overall survival (OS) and progression-free survival (PFS) times of patients according to serum miRNA levels. To define high and low serum miRNA levels, we used a cut-off that corresponded to the mean values of each miRNA in the serum of HDs plus 2 standard deviations. MiRNA levels were scored as low when below the cut-off and high when above the cut-off. The survival time was calculated from the date of sample collection at the beginning of the study. A log-rank test was used to analyze the differences between groups. The association and survival analyses were performed using SPSS version 19 software for Windows (SPSS, Chicago, IL). P values <0.05 (2-tailed) were considered statistically significant.

## Results

### MiR-192 as endogenous reference in the serum of HDs and breast cancer patients

**Figure 1:**
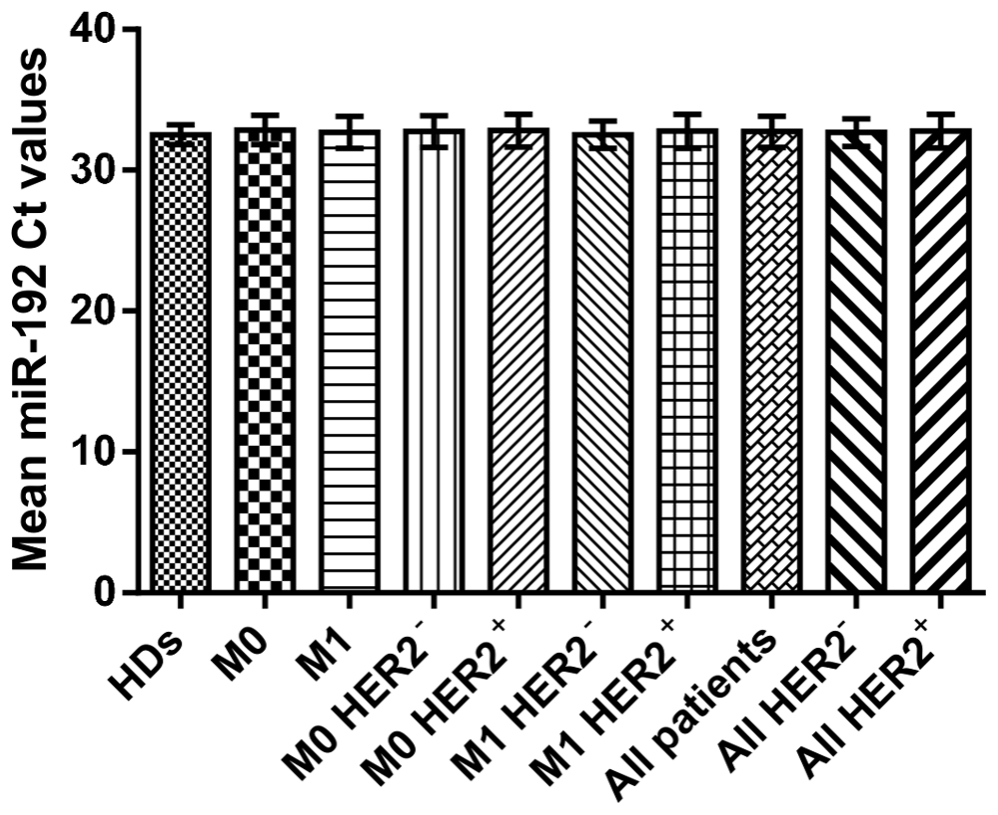
Mean threshold cycle values of miR-192± standard deviation in the serum of breast cancer patients and healthy donors. No significant differences in mean threshold cycle (Ct) values of miR-192 were observed among the serum of different groups (Kruskal–Wallis test, p = 0.785).

To determine the levels of serum miRNAs, it was necessary to select a normalizer with stable levels in the serum of HDs and patients. Because it was recently reported that the serum levels of miR-16, the endogenous control commonly used for qRT-PCR normalization, can be significantly affected by red blood cell hemolysis [33], we used an alternative normalizer. Previously, Vasilescu C *et al*. [34] reported that miR-192 was a reliable endogenous control for evaluating miRNA levels in the plasma of sepsis patients. Thus, we determined whether miR-192 was a reliable endogenous control also in breast cancer patients. The mean Ct values of miR-192 were constant, reproducible, and similar in the serum of breast cancer patients and HDs, with no statistically significant differences across all the serum samples (Figure 1, Table 2). To further confirm the reliability of miR-192 as endogenous control, we assessed the difference in the qRT-PCR normalization of serum miRNA levels between miR-192 and the commonly used miR-16. We calculated the fold difference in the levels of miR-21, miR-19a and miR-10b between the serum of breast cancer patients and HDs using the 2^−ΔΔCT^ method and compared the results using miR-192 and miR-16 as endogenous controls. As shown in figure S1, the fold differences in the levels of the three serum miRNAs were comparable using miR-192 and miR-16 as endogenous controls.

**Table 2:** Mean threshold cycle values of miR-192± standard deviation and 95% confidence interval in the serum of breast cancer patients and healthy donors.

	miR-192			miR-192		
Patients (n)	(mean Ct ± SD)	(95% CI)	Patients (n)	(mean Ct ± SD)	(95% CI)^b^	p value
HER2^−^ (48)	32.68±0.98	32.39–3.96	HER2^+^ (65)	32.78±1.20	32.48–33.08	0.486
M0 HER2^−^ (21)	32.99±0.89	32.58–33.41	M0 HER2^+^ (14)	32.81±1.17	32.13–33.48	0.958
M1 HER2^−^ (27)	32.52±0.95	32.14–32.90	M1 HER2^+^ (51)	32.77±1.22	32.43–33.11	0.226
M0 (35)	32.85±1.05	32.49–33.21	M1 (78)	32.68±1.13	32.43–32.94	0.533
All patients (113)	32.74±1.11	32.53–32.94	HDs (30)	32.51±0.69	32.25–32.77	0.114

Unpaired *t*-test (Mann-Whitney U test).

SD: standard deviation; CI: confidence interval; Ct: threshold cycle.

On the basis of this observation, we concluded that miR-192 could be used to normalize the levels of miRNAs in the serum of breast cancer patients and HDs.

### Serum miR-21, miR-10b, and miR-19a levels in M0 breast cancer patients

**Figure 2:**
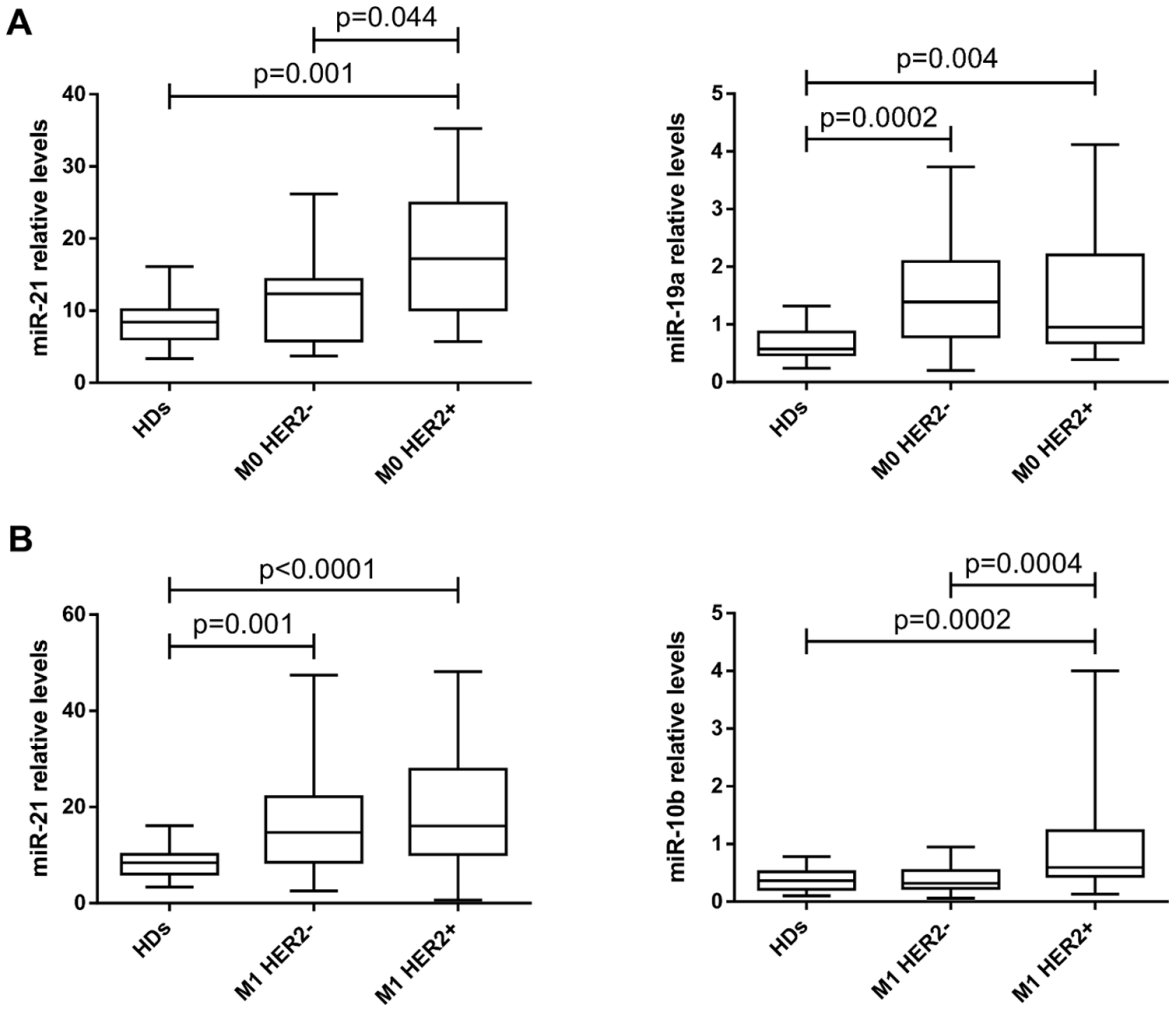
Serum miRNA levels in patients with M0 HER2^+^, M0 HER2^−^, M1 HER2^+^, M1 HER2^−^ breast cancer and HDs. The box plots show: a) relative serum miR-21 and miR-19a levels in patients with M0 HER2^+^ and M0 HER2^−^ breast cancer; and b) relative serum miR-21 and miR-10b levels in patients with M1 HER2^+^ and M1 HER2^−^ breast cancer. Thirty HDs were included as a control group. The differences in serum levels were evaluated using the Mann-Whitney U test, and the p values are indicated above the plots.

We evaluated miR-21, miR-10b, and miR-19a levels in the serum of M0 patients according to HER2-overexpression or amplification status and IBC type (Table S1). We found no significant differences in the median levels of the three serum miRNAs between IBC and non-IBC patients (data not shown). Patients with HER2^+^ breast cancer had higher serum miR-21 median levels than patients with HER2^−^ breast cancer (17.22 vs. 12.37, p = 0.044) and HDs (17.22 vs. 8.40, p = 0.001). Patients with HER2^+^ (0.95 vs. 0.57, p = 0.004) and HER2^−^ (1.39 vs. 0.57, p = 0.0002) breast cancer had higher serum miR-19a median levels than HDs; however, there were no significant differences between patients with HER2^+^ and HER2^−^ breast cancer (Figure 2A). We also found no significant differences in serum miR-10b median levels among patients with HER2^+^ and HER2^−^ breast cancer and HDs (Figure S2). Of note, patients with HER2^+^ breast cancer had 2-fold higher serum miR-21 median levels than HDs; whereas patients with HER2^−^ breast cancer had 2.4-fold higher serum miR-19a median levels than HDs (Table S1). The ROC curve analysis demonstrated that in M0 patients, serum miR-19a levels could differentiate patients with HER2^−^ (AUC  = 0.814; p = 0.0001) and HER2^+^ (AUC  = 0.774; p = 0.004) breast cancer from HDs; whereas serum miR-21 levels could distinguish patients with HER2^+^ from patients with HER2^−^ breast cancer (AUC  = 0.707; p = 0.042) and HDs (AUC  = 0.812; p = 0.001) (Figure S3).

### Serum miR-21, miR-10b, and miR-19a levels in M1 breast cancer patients

We evaluated miR-21, miR-10b, and miR-19a levels in the serum of M1 patients according to HER2-overexpression or amplification status and IBC type (Table S1). There were no significant differences in serum miR-21 and miR-10b median levels between patients with M1 IBC (MIBC) and M1 non-IBC (MNIBC) (data not shown). Patients with HER2^+^ and HER2^−^ breast cancer had higher serum miR-21 median levels than HDs (16.08 vs. 8.40, p<0.0001; and 14.37 vs. 8.40, p = 0.001, respectively). On the other hand, patients with HER2^+^ breast cancer had higher serum miR-10b median levels than patients with HER2^−^ breast cancer (0.59 vs. 0.32, p = 0.0004) and HDs (0.59 vs. 0.36, p = 0.0002) (Figure 2B). Of note, patients with HER2^+^ breast cancer had 1.9-fold higher serum miR-21 median levels than HDs (16.08 vs. 8.40) (Table S1). Moreover, the ROC curve analysis revealed that serum miR-21 levels could distinguish patients with HER2^−^ (AUC  = 0.763, p = 0.001) and HER2^+^ (AUC  = 0.804, p<0.0001) breast cancer from HDs; whereas serum miR-10b levels could distinguish patients with HER2^+^ from patients with HER2^−^ breast cancer (AUC  = 0.749; p = 0.0003) and HDs (AUC  = 0.756; p = 0.0001) (Figure S4).

**Figure 3:**
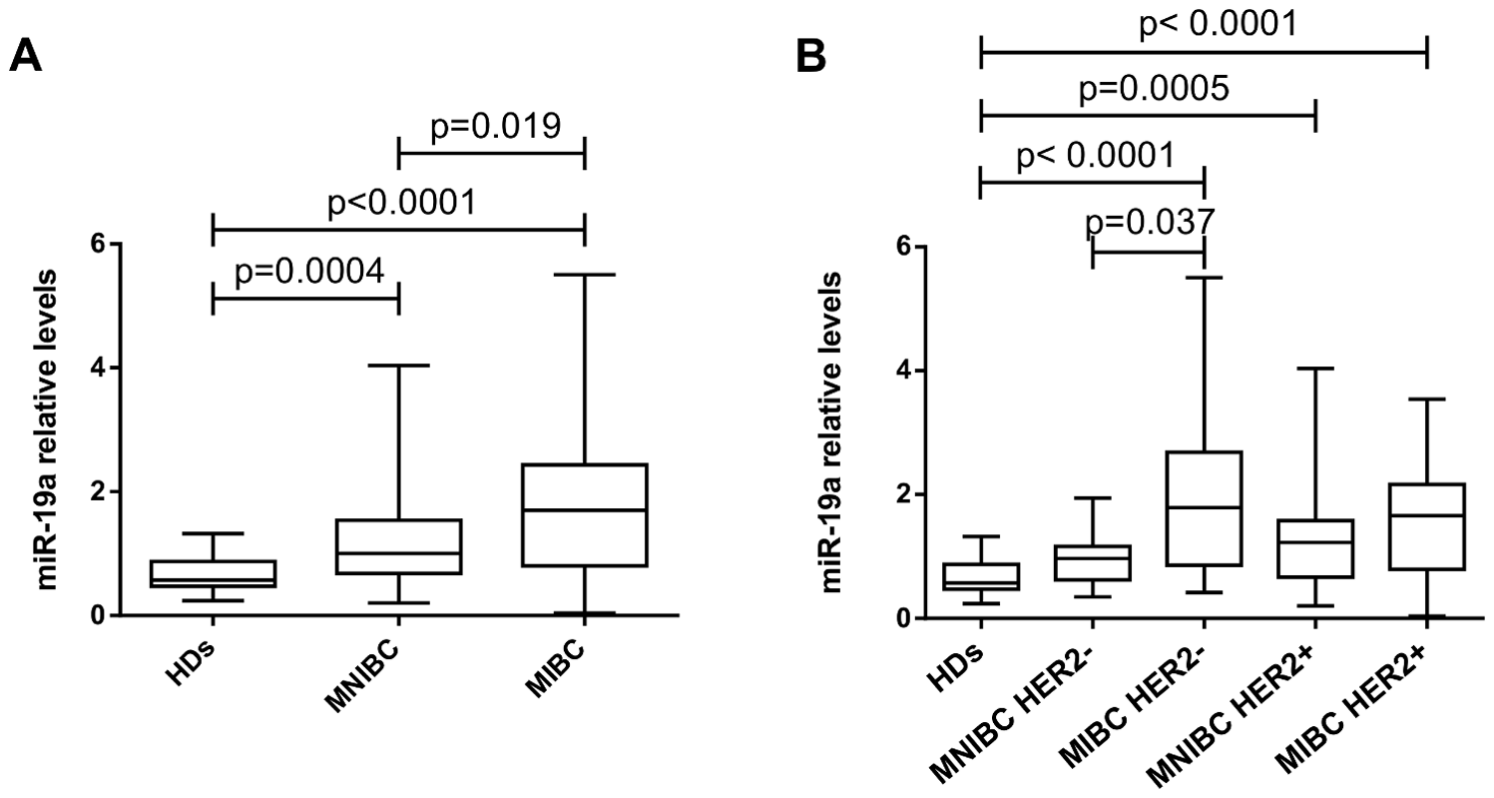
Serum miR-19a levels in patients with MIBC and MNIBC. The box plots show: a) relative serum miR-19a levels in patients with MNIBC and MIBC; and b) relative serum miR-19a levels in patients with MNIBC HER2^−^, MIBC HER2^−^, MNIBC HER2^+^ and MIBC HER2^+^. Thirty HDs were included as controls. The differences in serum miR-19a levels were evaluated using the Mann-Whitney U test, and the p values are indicated above the plots.

Patients with MIBC had significantly higher serum miR-19a median levels than patients with MNIBC (1.70 vs. 1.00, p = 0.019) (Figure 3A). Particularly, patients with MIBC HER2^−^ had significantly higher serum miR-19a median levels than patients with MNIBC HER2^−^ (1.79 vs. 0.96, p = 0.037) (Figure 3B). In addition, patients with MIBC HER2^+^ had higher serum miR-19a median levels than patients with MNIBC HER2^+^ (1.66 vs. 1.22), but this difference was not statistically significant. Serum miR-19a median levels were also higher in patients with MIBC HER2^−^, MNIBC HER2^+^, and MIBC HER2^+^ than in HDs (p<0.0001; p = 0.0005 and p<0.0001, respectively) (Figure 3B) and were 3.1-, 2.1-, and 2.9-fold higher than in HDs, respectively (Table S1). Then, we determined whether serum miR-19a levels could distinguish between patients with IBC and non-IBC. We found that serum miR-19a levels could distinguish patients with MIBC HER2^−^ from patients with MNIBC HER2^−^ (AUC  = 0.747; p = 0.035) (Figure S5A); however it had weak discriminatory power in the comparison between patients with MIBC HER2^+^ and MNIBC HER2^+^ (AUC  = 0.607: p = 0.190) (Figure S5B). Furthermore, serum miR-19a levels could distinguish patients with MIBC HER2^−^ (AUC  = 0.846; p<0.0001), MNIBC HER2^+^ (AUC  = 0.778; p = 0.0005), and MIBC HER2^+^ (0.825; p<0.0001) from HDs (Figure S6).

### Serum miRNA levels and clinicopathologic variables

To determine whether serum miR-21, miR-10b, and miR-19a levels were associated with clinicopathologic variables, we correlated them with prognostic factors (Table 1). High serum miR-19a levels were significantly associated with IBC type (p = 0.039) and the triple receptor negative status (p = 0.037). A significant association was also observed between high serum miR-10b levels and HER2-overexpression or amplification (p = 0.001) and stage (p = 0.034).

**Figure 4:**
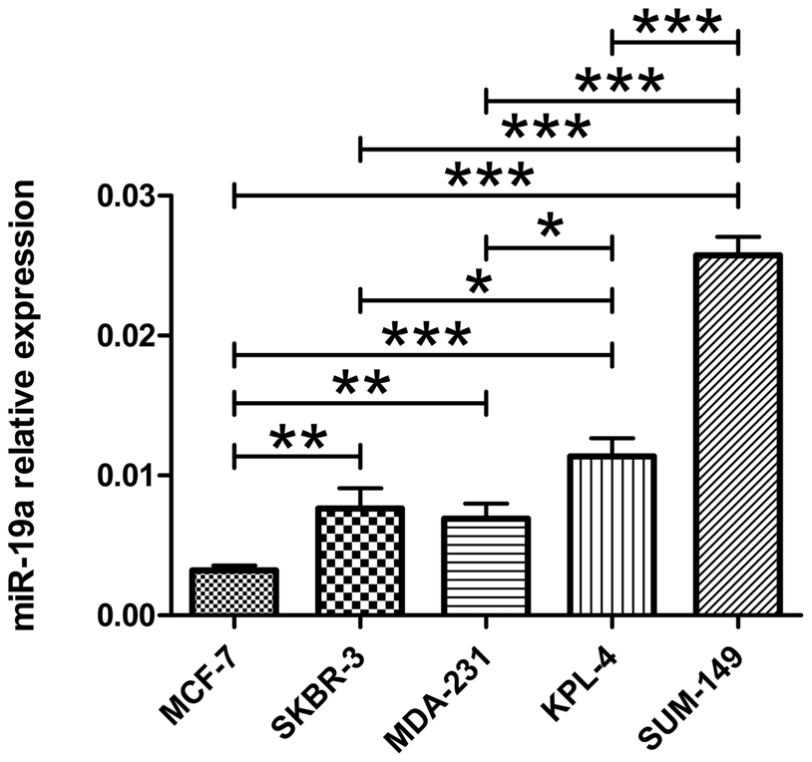
MiR-19a expression in breast cancer cell lines. The expression of miR-19a was evaluated in 5 breast cancer cell lines. The two IBC cell lines SUM-149 (TN) and KPL-4 (HER2^+^) expressed significantly higher levels of miR-19a than the non-IBC cell lines MCF-7 (ER^+^), SKBR-3 (HER2^+^), and MDA-231 (TN) (mean ± standard deviation; 2-tailed Student's *t-*test; p<0.05 is indicated with: *; p<0.01 is indicated with: **; p<0.001 is indicated with: ***).

Because high serum miR-19a levels were associated with IBC, we determined whether IBC tumor cells could contribute to the increased miR-19a levels in the serum of IBC patients. We evaluated miR-19a expression in MCF-7, SKBR-3, KPL-4, MDA-231, and SUM-149 breast cancer cells and we found that the two IBC cell lines, SUM-149 and KPL-4, had significantly higher expression levels of miR-19a than the non-IBC cell lines MCF-7, SKBR-3 and MDA-231 (Figure 4). Therefore, IBC tumor cells may contribute to the increased miR-19a levels in the serum of IBC patients.

### Serum miRNA levels and clinical outcome

We determined whether serum miR-21, miR-10b, and miR-19a levels, measured at the beginning of the study, were predictive of patients' outcome.

**Figure 5:**
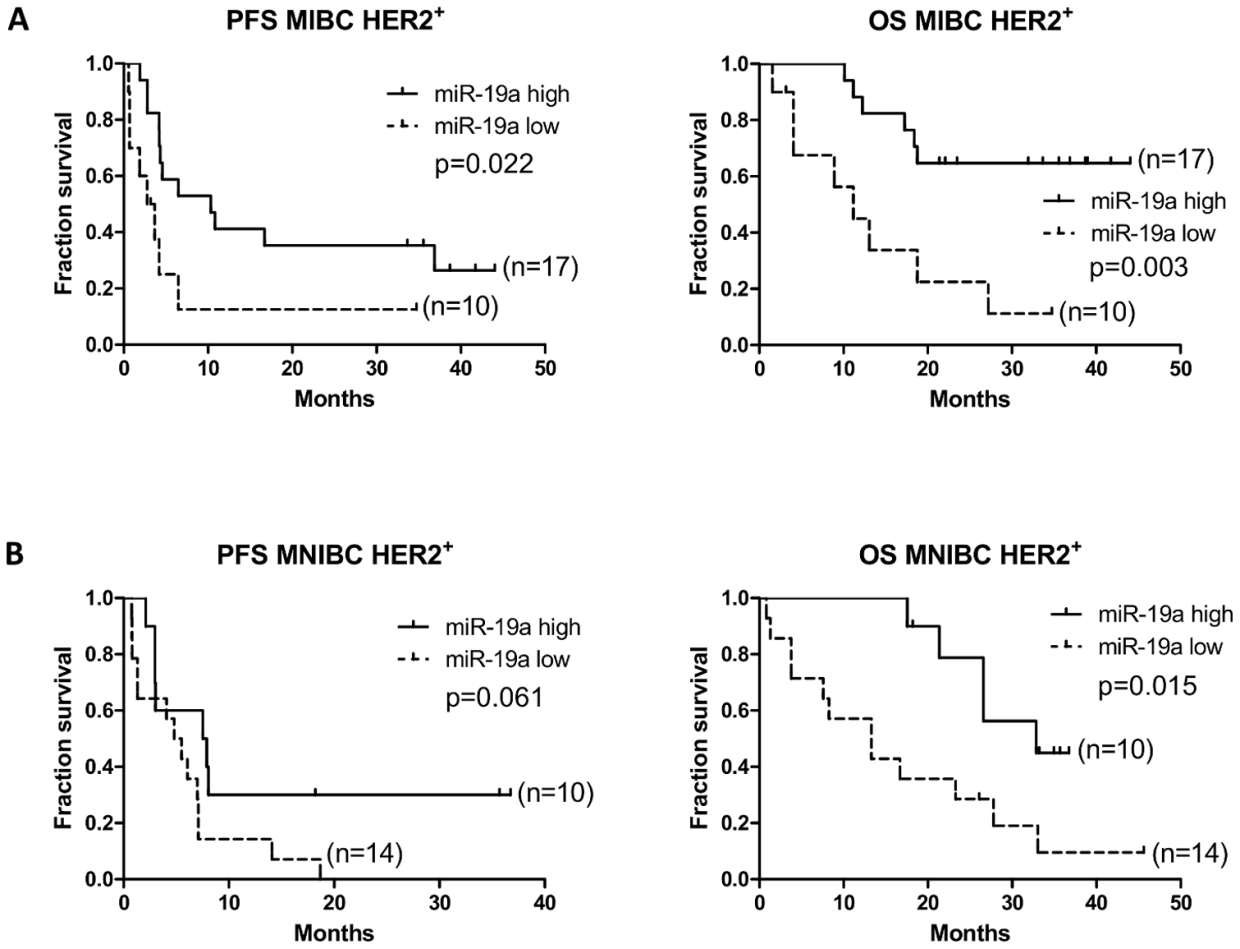
Kaplan-Meier plots of breast cancer patients according to serum miR-19a levels. The Kaplan-Meier plots show the survival time of breast cancer patients according to serum miR-19a levels. In a) patients with MIBC HER2^+^ and high serum miR-19a levels had longer PFS time (10.3 vs. 3.2 months; p = 0.022) and OS time (median not reached vs. 11.2 months; p = 0.003) than patients with MIBC HER2^+^ and with low serum miR-19a levels; in b) patients with MNIBC HER2^+^ and high serum miR-19a levels had longer PFS time (7.7 vs. 5.1 months; p = 0.061) and OS time (32.9 vs. 13.3 months; p = 0.015) than patients with MNIBC HER2^+^ and low serum miR-19a levels. High and low serum miR-19a levels were defined according to a cut-off corresponding to the mean values of miR-19a in the serum of HDs plus 2 standard deviations. A log-rank test was used to analyze the differences in the survival times between patients with high and low serum miR-19a levels. Characteristics of patients with MIBC HER2^+^ and high serum miR-19a levels: median age (48.3); trastuzumab-treated (12/17 = 70.6%); treatment-naïve (3/17 = 17.6%); no trastuzumab-treated (2/17 = 11.8%). Characteristics of patients with MIBC HER2^+^ and low serum miR-19a levels: median age (53.2); trastuzumab-treated (5/10 = 50.0%); treatment-naïve (0/10 = 0.0%); no trastuzumab-treated (5/10 = 50.0%). Characteristics of patients with MNIBC HER2^+^ and high serum miR-19a levels: median age (53.8); trastuzumab-treated (5/10 = 50.0%); treatment-naïve (2/10 = 20.0%), no trastuzumab-treated (3/10 = 30.0%). Characteristics of patients with MNIBC HER2^+^ and low serum miR-19a levels: median age (54.1); trastuzumab-treated (11/14 = 78.6%); treatment-naïve (1/14 = 7.1%); no trastuzumab-treated (2/14 = 14.3%).

In the M0 cohort, we found no significant differences in the survival outcomes between patients with high and low levels of the three serum miRNAs (data not shown). On the other hand, in the M1 cohort, patients with MIBC HER2^+^ and high serum miR-19a levels at the beginning of the study had longer PFS time (10.3 vs. 3.2 months; p = 0.022) and OS time (median not reached vs. 11.2 months; p = 0.003) than patients with MIBC HER2^+^ and low serum miR-19a levels (Figure 5A). A similar survival pattern was observed in MNIBC patients. In particular, patients with MNIBC HER2^+^ and high serum miR-19a levels had longer but not statistical significant PFS time (7.7 vs. 5.1 months; p = 0.061) and statistically significant longer OS time (32.9 vs. 13.3 months; p = 0.015) than patients with MNIBC HER2^+^ and low serum miR-19a levels (Figure 5B). Interestingly, there were no significant differences in survival times in both patients with MIBC and MNIBC HER2^−^ according to serum miR-19a levels (not shown). In M1 cohort, there were also no significant differences in PFS and OS times according to serum miR-21 and miR-10b levels (not shown).

## Discussion

Over the past few years, several studies on circulating miRNAs in breast cancer used miR-16 as an endogenous control to normalize qRT-PCR data [35]–[38]. Recently, miR-16 was found to be highly expressed by red blood cells, and its serum and plasma levels increased significantly in hemolyzed samples [33]. Therefore, sample alteration during the collection and processing procedure may affect miR-16 serum levels. In our study, we proposed and validated miR-192 as an alternative endogenous control for evaluating miRNA serum levels in breast cancer patients. To our knowledge, this is the first report of miR-192 being used for qRT-PCR normalization of serum miRNAs levels in breast cancer patients; previously, it was used to evaluate the prognostic value of plasma miRNAs in patients with sepsis [34].

MiR-21, miR-10b, and miR-19a overexpression endows breast cancer cells with more aggressive behavior, such as increased proliferation, migration, invasion, neoangiogenesis, apoptosis resistance, resulting in disease progression via tumor cell dissemination and metastasis formation [8], [22]–[31]. As levels of serum miRNAs may reflect the clinicopathologic status of cancer patients and correlate with the dysregulated pattern of the parental tumor, in this study we assessed if the levels of miR-21, miR-10b, and miR-19a in the serum of breast cancer patients were associated with aggressive characteristics of tumor cells (HER2-overexpression or amplification and IBC type), and if they had prognostic value.

In the M0 cohort, we found that serum miR-21 median levels were significantly higher in patients with HER2^+^ than in patients with HER2^−^ breast cancer and HDs and this difference could distinguish patients with HER2^+^ breast cancer from patients with HER2^−^ breast cancer and HDs. In this regard, it has been shown that HER2 signaling can upregulate miR-21 expression in breast cancer cells [39]. Therefore, it can be hypothesized that increased HER2 signaling in patients with HER2^+^ breast cancer can enhance miR-21 expression in tumor cells and subsequently contribute to the increased serum miR-21 levels by being released into the extracellular space. We also found that patients with HER2^+^ and HER2^−^ breast cancer had significantly higher serum miR-19a median levels than HDs; thus, serum miR-19a levels could distinguish breast cancer patients from HDs. As miR-19a regulates tumor cell survival by reducing apoptosis [28], it can be hypothesized that, higher miR-19a serum levels in M0 breast cancer patients might, in part, derive from tumor cells with increased survival ability. However, we found no statistically significant differences in patients' survival according to serum miR-21 and miR-19a levels. We also found no significant differences in patients' serum miR-10b levels and in the levels of the three serum miRNAs between patients with IBC and non-IBC. It should be noticed that 30 of 34 M0 patients (97.1%) were newly diagnosed; thus, the serum miRNA levels may represent the clinicopathologic disease characteristics at the beginning of the study, as they were not affected by previous or concurrent treatments. Indeed, anti-tumor treatment may cause an increase in the serum levels of tumor-derived miRNAs by treatment-induced apoptosis and necrosis.

In the M1 cohort, we found significant differences in serum miR-10b levels between patients with HER2^+^ and HER2^−^ breast cancer. In a previous report from our laboratory, Giordano A *et al*. [32] revealed that 76.5% of M1 patients with HER2^+^ breast cancer had circulating tumor cells with epithelial-to-mesenchymal (EMT) characteristics that expressed the transcription factor Twist1. Besides regulating EMT, Twist1 is also a transcription factor for miR-10b [27]; thus, it can be hypothesized that tumor cells with EMT characteristics may contribute to the increased miR-10b levels in the serum of M1 patients with HER2^+^ breast cancer. However, we found no differences in patient's survival according to serum miR-10b levels. In the M1 cohort, patients with HER2^+^ and HER2^−^ breast cancer had significantly higher serum miR-21 median levels than HDs, and patients with HER2^+^ breast cancer had higher serum miR-21 median levels than patients with HER2^−^ breast cancer (Table S1), but that difference was not statistically significant. We found no differences in patients' survival according to serum miR-21 levels.

To our knowledge, our study is the first to find an association between high levels of serum miR-19a and IBC. In particular, patients with MIBC had significantly higher levels of serum miR-19a than patients with MNIBC, and serum miR-19a could distinguish between these two patients' groups. We also found that high levels of miR-19a were expressed in the two IBC cell lines, KPL-4 and SUM-149 compared with the non-IBC cell lines MCF-7, SKBR-3, and MDA-231. Therefore, IBC cells that express high levels of miR-19a, may contribute to the increased miR-19a levels in the serum of patients with MIBC. It should be noted that tumor cells can release miRNAs either by active secretion or cell lysis/apoptosis upon cell death induced by anti-tumor treatment. In our study, most M1 patients (64 of 75 [85.3%]) underwent treatment before the beginning of this study; thus, increased serum miRNA levels in patients with good prognosis may be due to treatment-induced death of sensitive tumor cells.

Besides tumor cells, other cell types may contribute to serum miRNA levels. Of note, most circulating miRNAs originate from blood cells and the same miRNAs can be expressed also by tumor cells, as reported recently by Pritchard C *et al*. [33]. Therefore, it can be difficult to distinguish between the individual contribution of blood and tumor cells to serum miRNA levels. MiR-19a expression is increased in T lymphocytes upon activation. In particular, both activated antigen-specific effector CD8^+^ lymphocytes [40] and T helper-1 lymphocytes (Th1) [41] upregulate the expression of miR-19a. Th1 cells play an important role in the activation of cell-mediated anti-tumor immunity of antigen-specific CD8^+^ T cytotoxic lymphocytes (CD8^+^ CTL) and NK cells. As activated lymphocytes can secrete miRNAs [42], the induction of a competent anti-tumor immune response may contribute to the increased serum miR-19a levels in patients with good prognosis. Furthermore, trastuzumab can mediate antibody-dependent cellular cytotoxicity (ADCC) resulting in the tumor cell lysis/apoptosis induced by the natural killer (NK) cells, the principal immune cells involved in ADCC [43]. Effective activation of Th1 cells can enhance NK-mediated ADCC and accordingly the clinical efficacy of trastuzumab. Therefore, the high levels of miR-19a in the serum of patients with HER2^+^ breast cancer who received anti-HER2 therapy and had good prognosis may result from an effective Th1-mediated immune response that enhanced NK-mediated tumor cell lysis in ADCC. On the other hand, patients with HER2^+^ breast cancer and worse prognosis may have a poor response to trastuzumab therapy do to an ineffective Th1-mediated immune response and accordingly a reduced NK-mediated tumor cell lysis resulting in reduced levels of serum miR-19a. Preliminary data from our laboratory support these results by showing that patients with IBC with higher percentage of Th1 cells in peripheral blood cells had better prognosis than patients with lower percentage of Th1 cells (unpublished data). The ADCC may also, in part, explain the higher levels of miR-10b and miR-21 in patients with HER2^+^ compared with patients with HER2^−^ breast cancer. However, miR-10b and miR-21 could not distinguish between patients with good and poor prognoses.

Very recently, it was reported that levels of serum miR-19a correlated with worse prognosis in patients with non-small cell lung cancer (NSCLC) and increased serum miR-19a levels may reflect aggressive characteristics of NSCLC tumor cells [44]. In this study, all the serum samples were collected before treatment; thus levels of serum miR-19a were not affected by treatment. In our laboratory, we found that, in the SUM-149 cell line, the acquisition of aggressive phenotype correlated with increased expression of miR-19a (unpublished data). Therefore, IBC HER2^−^ cells with aggressive characteristics may secrete higher levels of miR-19a. In M0 cohort, we could not find a significant difference in the survival of treatment naïve patients according to serum miR-19a levels due to the small number of patients. However, in the M1 cohort, we found that although patients with MIBC HER2^+^ had comparable serum miR-19a levels with patients with MIBC HER2^−^ (Figure 3B), patients with MIBC HER2^+^ had a longer OS time (27.2 vs. 16.1 months; p = 0.014) (Figure S7). Therefore, in patients with MIBC HER2^−^, the high serum miR-19a levels may be due to the secretion from treatment-resistant tumor cells; whereas in patients with MIBC HER2^+^, the high serum miR-19a levels may be due to an effective Th1-mediated immune response and cells lysis/apoptosis of treatment-sensitive tumor cells (ADCC).

A weakness of our study is the relative small size of the number of patients in each group; therefore, our results need to be confirmed in an independent study with a larger number of patients. Furthermore, we need to confirm the contribution of breast cancer and immune cells to serum miRNAs in an *in vitro* model. In particular, more studies on miRNA release mechanisms (secretion, apoptosis, and necrosis) are required. We are currently evaluating the role of Th1 cells and NK cells in their contribution to the increased serum miR-19a levels.

In conclusion, higher levels of miR-21 and miR-10b were present in the serum of patients with M0 and M1 HER2^+^ breast cancer, respectively, compared with stage-matched patients with HER2^−^ breast cancer. High serum miR-19a levels were associated with IBC, represented a prognostic biomarker for favorable clinical outcome in patients with metastatic HER2^+^ breast cancer and could be associated to an effective immune cell-mediated anti-tumor response.

## Supporting Information

Figure S1 **Comparison between the two endogenous controls miR-192 and miR-16 in the qRT-PCR normalization of serum miRNA levels.** To validate the use of miR-192 in the qRT-PCR normalization of serum miRNAs, miR-192 and miR-16 were compared as endogenous controls. The equation 2^-ΔΔCt^ was used to calculate the fold difference of relative serum miRNAs levels between breast cancer patients and HDs. No significant differences were measured between miR-192 and miR-16 when used as endogenous controls (Wilcoxon matched-pairs signed rank). Fifteen HDs and thirty-eight patients'serum samples were included for the comparison. The mean Ct and standard deviation of miR-16 in HDs' and patients' serum samples were 30.20±1.01 and 29.57±0.97, respectively.
(TIF)

Figure S2 **Serum miR-10b levels in patients with M0 HER2^+^, M0 HER2^−^ breast cancer and HDs.** The box plots show no significant difference in the serum miR-10b levels of patients with M0 HER2^+^ and M0 HER2^−^ breast cancer and HDs. Thirty HDs were included as a control group. The differences in serum miR-10b levels were evaluated using the Mann-Whitney U test.
(TIF)

Figure S3 **ROC curve analysis of serum miRNAs in patients with M0 breast cancer.** The ROC curve analysis shows the ability of serum miR-19a levels to distinguish patients with M0 HER2^−^ (AUC  = 814; p = 0.0001) and M0 HER2^+^ (AUC  = 0.774; p = 0.004) breast cancer from HDs. Serum miR-21 levels could distinguish patients with M0 HER2^+^ from patient with M0 HER2^−^ breast cancer (AUC  = 0.707; p = 0.042) and HDs (AUC  = 0.812; p = 0.001)
(TIF)

Figure S4 **ROC curve analysis of serum miRNAs in patients with M1 breast cancer.** The ROC curve analysis shows the ability of serum miR-21 levels to distinguish patient with M1 HER2^−^ (AUC  = 0.763, p = 0.001) and M1 HER2^+^ (AUC  = 0.804, p<0.0001) breast cancer from HDs. Serum miR-10b levels could distinguish patients with M1 HER2^+^ from patients with M1 HER2^−^ breast cancer (AUC  = 0.749; p = 0.0003) and HDs (AUC  = 0.756 p  = 0.0001).
(TIF)

Figure S5 **ROC curve analysis of serum miR-19a in patients with MNIBC HER2^−^, MIBC HER2^−^, MNIBC HER2^+^ and MIBC HER2^+^ breast cancer.** The ROC curve analysis shows: a) serum miR-19a levels could distinguish between patients with MIBC HER2^−^ from patients with MNIBC HER2^−^ patients (AUC  = 0.747; p = 0.035); and b) serum miR-19a levels had low power for distinguishing between patients with MIBC HER2^+^ from patients with MNIBC HER2^+^ breast cancer (AUC  = 0.607; p = 0.190).
(TIF)

Figure S6 **ROC curve analysis of serum miR-19a in patients with MIBC HER2** ^−^ **, MNIBC HER2^+^ and MIBC HER2^+^ breast cancer.** The ROC curve analysis shows that serum miR-19a levels could distinguish between patients with MIBC HER2^−^ (AUC = 0.846; p <0.0001), MNIBC HER2^+^ (AUC  = 0.778; p = 0.0005), MIBC HER2^+^ (0.825; p <0.0001) breast cancer and HDs.
(TIF)

Figure S7 **Overall survival in patients with MIBC HER2^+^ and MIBC HER2^−^.** Patients with MIBC HER2^+^ had similar levels of serum miR-19a compared with patients with MIBC HER2^−^ (1.66 vs. 1.79, respectively). However patients with MIBC HER2^+^ had longer OS time than patients with MIBC HER2^−^ (27.2 vs. 16.1 months; p = 0.014).
(TIF)

Table S1 **Serum miR-21, miR-10b, and miR-19a median levels in breast cancer patients and healthy donors.**
(DOCX)

## References

[1] Jemal A, Siegel R, Xu J, Ward E (2010) Cancer statistics, 2010. CA Cancer J Clin 60: 277–300. 20610543 10.3322/caac.20073

[2] Anderson WF, Schairer C, Chen BE, Hance KW, Levine PH (2005) Epidemiology of inflammatory breast cancer (IBC). Breast Dis 22: 9–23. 16735783 10.3233/bd-2006-22103 PMC2852616

[3] Kleer CG, van Golen KL, Merajver SD (2000) Molecular biology of breast cancer metastasis. Inflammatory breast cancer: clinical syndrome and molecular determinants. Breast Cancer Res 2: 423–429. 11250736 10.1186/bcr89 PMC138665

[4] Cristofanilli M, Valero V, Buzdar AU, Kau SW, Broglio KR, et al (2007) Inflammatory breast cancer (IBC) and patterns of recurrence: understanding the biology of a unique disease. Cancer 110: 1436–1444. 17694554 10.1002/cncr.22927

[5] Mariani G, Fasolo A, De Benedictis E, Gianni L (2009) Trastuzumab as adjuvant systemic therapy for HER2-positive breast cancer. Nat Clin Pract Oncol 6: 93–104. 19107109 10.1038/ncponc1298

[6] Nahta R, Yu D, Hung MC, Hortobagyi GN, Esteva FJ (2006) Mechanisms of disease: understanding resistance to HER2-targeted therapy in human breast cancer. Nat Clin Pract Oncol 3: 269–280. 16683005 10.1038/ncponc0509

[7] Calin GA, Sevignani C, Dumitru CD, Hyslop T, Noch E, et al (2004) Human microRNA genes are frequently located at fragile sites and genomic regions involved in cancers. Proc Natl Acad Sci U S A 101: 2999–3004. 14973191 10.1073/pnas.0307323101 PMC365734

[8] Iorio MV, Ferracin M, Liu CG, Veronese A, Spizzo R, et al (2005) MicroRNA gene expression deregulation in human breast cancer. Cancer Res 65: 7065–7070. 16103053 10.1158/0008-5472.CAN-05-1783

[9] Lawrie CH, Gal S, Dunlop HM, Pushkaran B, Liggins AP, et al (2008) Detection of elevated levels of tumour-associated microRNAs in serum of patients with diffuse large B-cell lymphoma. Br J Haematol 141: 672–675. 18318758 10.1111/j.1365-2141.2008.07077.x

[10] Mitchell PS, Parkin RK, Kroh EM, Fritz BR, Wyman SK, et al (2008) Circulating microRNAs as stable blood-based markers for cancer detection. Proc Natl Acad Sci U S A 105: 10513–10518. 18663219 10.1073/pnas.0804549105 PMC2492472

[11] Ng EK, Chong WW, Jin H, Lam EK, Shin VY, et al (2009) Differential expression of microRNAs in plasma of patients with colorectal cancer: a potential marker for colorectal cancer screening. Gut 58: 1375–1381. 19201770 10.1136/gut.2008.167817

[12] Hu Z, Chen X, Zhao Y, Tian T, Jin G, et al (2010) Serum microRNA signatures identified in a genome-wide serum microRNA expression profiling predict survival of non-small-cell lung cancer. J Clin Oncol 28: 1721–1726. 20194856 10.1200/JCO.2009.24.9342

[13] Taylor DD, Gercel-Taylor C (2008) MicroRNA signatures of tumor-derived exosomes as diagnostic biomarkers of ovarian cancer. Gynecol Oncol 110: 13–21. 18589210 10.1016/j.ygyno.2008.04.033

[14] van Schooneveld E, Wouters MC, Van der Auwera I, Peeters DJ, Wildiers H, et al (2012) Expression profiling of cancerous and normal breast tissues identifies microRNAs that are differentially expressed in serum from patients with (metastatic) breast cancer and healthy volunteers. Breast Cancer Res 14: R34. 22353773 10.1186/bcr3127 PMC3496152

[15] Brase JC, Wuttig D, Kuner R, Sultmann H (2010) Serum microRNAs as non-invasive biomarkers for cancer. Mol Cancer 9: 306. 21110877 10.1186/1476-4598-9-306 PMC3002336

[16] Valadi H, Ekstrom K, Bossios A, Sjostrand M, Lee JJ, et al (2007) Exosome-mediated transfer of mRNAs and microRNAs is a novel mechanism of genetic exchange between cells. Nat Cell Biol 9: 654–659. 17486113 10.1038/ncb1596

[17] Zernecke A, Bidzhekov K, Noels H, Shagdarsuren E, Gan L, et al (2009) Delivery of microRNA-126 by apoptotic bodies induces CXCL12-dependent vascular protection. Sci Signal 2: ra81. 19996457 10.1126/scisignal.2000610

[18] Cortez MA, Calin GA (2009) MicroRNA identification in plasma and serum: a new tool to diagnose and monitor diseases. Expert Opin Biol Ther 9: 703–711. 19426115 10.1517/14712590902932889

[19] Hunter MP, Ismail N, Zhang X, Aguda BD, Lee EJ, et al (2008) Detection of microRNA expression in human peripheral blood microvesicles. PLoS One 3: e3694. 19002258 10.1371/journal.pone.0003694 PMC2577891

[20] Arroyo JD, Chevillet JR, Kroh EM, Ruf IK, Pritchard CC, et al (2011) Argonaute2 complexes carry a population of circulating microRNAs independent of vesicles in human plasma. Proc Natl Acad Sci U S A 108: 5003–5008. 21383194 10.1073/pnas.1019055108 PMC3064324

[21] Chen X, Ba Y, Ma L, Cai X, Yin Y, et al (2008) Characterization of microRNAs in serum: a novel class of biomarkers for diagnosis of cancer and other diseases. Cell Res 18: 997–1006. 18766170 10.1038/cr.2008.282

[22] Si ML, Zhu S, Wu H, Lu Z, Wu F, et al (2007) miR-21-mediated tumor growth. Oncogene 26: 2799–2803. 17072344 10.1038/sj.onc.1210083

[23] Yan LX, Wu QN, Zhang Y, Li YY, Liao DZ, et al (2011) Knockdown of miR-21 in human breast cancer cell lines inhibits proliferation, in vitro migration and in vivo tumor growth. Breast Cancer Res 13: R2. 21219636 10.1186/bcr2803 PMC3109565

[24] Liu LZ, Li C, Chen Q, Jing Y, Carpenter R, et al (2011) MiR-21 induced angiogenesis through AKT and ERK activation and HIF-1alpha expression. PLoS One 6: e19139. 21544242 10.1371/journal.pone.0019139 PMC3081346

[25] Yan LX, Huang XF, Shao Q, Huang MY, Deng L, et al (2008) MicroRNA miR-21 overexpression in human breast cancer is associated with advanced clinical stage, lymph node metastasis and patient poor prognosis. RNA 14: 2348–2360. 18812439 10.1261/rna.1034808 PMC2578865

[26] Gong C, Yao Y, Wang Y, Liu B, Wu W, et al (2011) Up-regulation of miR-21 mediates resistance to trastuzumab therapy for breast cancer. J Biol Chem 286: 19127–19137. 21471222 10.1074/jbc.M110.216887 PMC3099726

[27] Ma L, Teruya-Feldstein J, Weinberg RA (2007) Tumour invasion and metastasis initiated by microRNA-10b in breast cancer. Nature 449: 682–688. 17898713 10.1038/nature06174

[28] Olive V, Bennett MJ, Walker JC, Ma C, Jiang I, et al (2009) miR-19 is a key oncogenic component of mir-17–92. Genes Dev 23: 2839–2849. 20008935 10.1101/gad.1861409 PMC2800084

[29] Mu P, Han YC, Betel D, Yao E, Squatrito M, et al (2009) Genetic dissection of the miR-17∼92 cluster of microRNAs in Myc-induced B-cell lymphomas. Genes Dev 23: 2806–2811. 20008931 10.1101/gad.1872909 PMC2800095

[30] Liu S, Goldstein RH, Scepansky EM, Rosenblatt M (2009) Inhibition of rho-associated kinase signaling prevents breast cancer metastasis to human bone. Cancer Res 69: 8742–8751. 19887617 10.1158/0008-5472.CAN-09-1541

[31] Dews M, Homayouni A, Yu D, Murphy D, Sevignani C, et al (2006) Augmentation of tumor angiogenesis by a Myc-activated microRNA cluster. Nat Genet 38: 1060–1065. 16878133 10.1038/ng1855 PMC2669546

[32] Giordano A, Gao H, Anfossi S, Cohen E, Mego M, et al (2012) Epithelial-mesenchymal transition and stem cell markers in patients with HER2-positive metastatic breast cancer. Mol Cancer Ther 11: 2526–2534. 22973057 10.1158/1535-7163.MCT-12-0460 PMC3500676

[33] Pritchard CC, Kroh E, Wood B, Arroyo JD, Dougherty KJ, et al (2012) Blood cell origin of circulating microRNAs: a cautionary note for cancer biomarker studies. Cancer Prev Res (Phila) 5: 492–497. 22158052 10.1158/1940-6207.CAPR-11-0370 PMC4186243

[34] Vasilescu C, Rossi S, Shimizu M, Tudor S, Veronese A, et al (2009) MicroRNA fingerprints identify miR-150 as a plasma prognostic marker in patients with sepsis. PLoS One 4: e7405. 19823581 10.1371/journal.pone.0007405 PMC2756627

[35] Zhao H, Shen J, Medico L, Wang D, Ambrosone CB, et al (2010) A pilot study of circulating miRNAs as potential biomarkers of early stage breast cancer. PLoS One 5: e13735. 21060830 10.1371/journal.pone.0013735 PMC2966402

[36] Heneghan HM, Miller N, Lowery AJ, Sweeney KJ, Newell J, et al (2010) Circulating microRNAs as novel minimally invasive biomarkers for breast cancer. Ann Surg 251: 499–505. 20134314 10.1097/SLA.0b013e3181cc939f

[37] Roth C, Rack B, Muller V, Janni W, Pantel K, et al (2010) Circulating microRNAs as blood-based markers for patients with primary and metastatic breast cancer. Breast Cancer Res 12: R90. 21047409 10.1186/bcr2766 PMC3046429

[38] Asaga S, Kuo C, Nguyen T, Terpenning M, Giuliano AE, et al (2011) Direct serum assay for microRNA-21 concentrations in early and advanced breast cancer. Clin Chem 57: 84–91. 21036945 10.1373/clinchem.2010.151845

[39] Huang TH, Wu F, Loeb GB, Hsu R, Heidersbach A, et al (2009) Up-regulation of miR-21 by HER2/neu signaling promotes cell invasion. J Biol Chem 284: 18515–18524. 19419954 10.1074/jbc.M109.006676 PMC2709372

[40] Wu T, Wieland A, Araki K, Davis CW, Ye L, et al (2012) Temporal expression of microRNA cluster miR-17-92 regulates effector and memory CD8+ T-cell differentiation. Proc Natl Acad Sci U S A 109: 9965–9970. 22665768 10.1073/pnas.1207327109 PMC3382487

[41] Sasaki K, Kohanbash G, Hoji A, Ueda R, McDonald HA, et al (2010) miR-17-92 expression in differentiated T cells – implications for cancer immunotherapy. J Transl Med 8: 17. 20167088 10.1186/1479-5876-8-17 PMC2836279

[42] Rechavi O, Erlich Y, Amram H, Flomenblit L, Karginov FV, et al (2009) Cell contact-dependent acquisition of cellular and viral nonautonomously encoded small RNAs. Genes Dev 23: 1971–1979. 19684116 10.1101/gad.1789609 PMC2725935

[43] Nahta R, Esteva FJ (2006) Herceptin: mechanisms of action and resistance. Cancer Lett 232: 123–138. 16458110 10.1016/j.canlet.2005.01.041

[44] Lin Q, Chen T, Lin G, Lin J, Chen G, et al (2013) Serum miR-19a expression correlates with worse prognosis of patients with non-small cell lung cancer. J Surg Oncol 107: 767–771. 23609137 10.1002/jso.23312

