# Supplementary figures and images for: High Serum miR-19a Levels Are Associated with Inflammatory Breast Cancer and Are Predictive of Favorable Clinical Outcome in Patients with Metastatic HER2+ Inflammatory Breast Cancer
Source: PLoS One. 2014 Jan 8;9(1):e83113. doi: 10.1371/journal.pone.0083113 (PMC3885405; doi:10.1371/journal.pone.0083113)

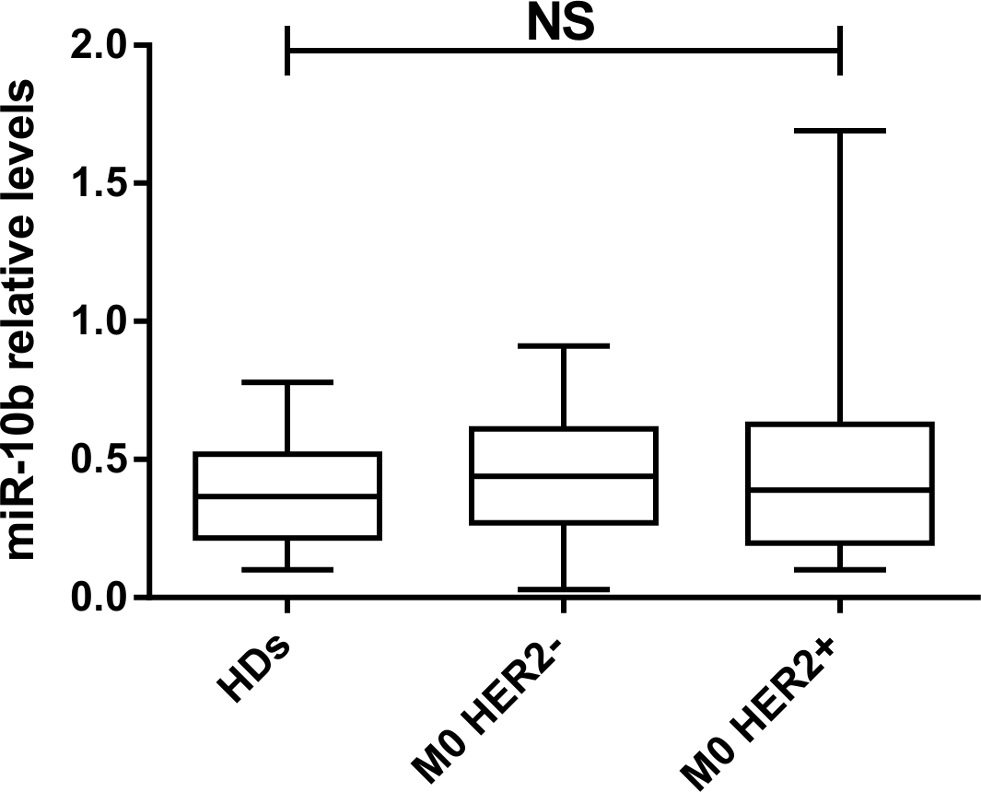

Supplement: Figure S2 — Serum miR-10b levels in patients with M0 HER2+, M0 HER2− breast cancer and HDs. The box plots show no significant difference in the serum miR-10b levels of patients with M0 HER2+ and M0 HER2− breast cancer and HDs. Thirty HDs were included as a control group. The differences in serum miR-10b levels were evaluated using the Mann-Whitney U test. (TIF) [file pone.0083113.s002.tif]
