# Supplementary material for: High Serum miR-19a Levels Are Associated with Inflammatory Breast Cancer and Are Predictive of Favorable Clinical Outcome in Patients with Metastatic HER2+ Inflammatory Breast Cancer
Source: PLoS One. 2014 Jan 8;9(1):e83113. doi: 10.1371/journal.pone.0083113 (PMC3885405; doi:10.1371/journal.pone.0083113)
Supplement: Table S1 — Serum miR-21, miR-10b, and miR-19a median levels in breast cancer patients and healthy donors. (DOCX) [file pone.0083113.s008.docx]

**Table S1.** Serum miR-21, miR-10b, and miR-19a median levels in breast cancer patients and healthy donors

| **Serum (n)** | **miR-21** | **miR-10b** | **miR-19a** |
| --- | --- | --- | --- |
| Healthy donors (30) | 8.40 | 0.36 | 0.57 |
| M0 (35) |  |  |  |
| HER2^-^ (21) | 12.37 | 0.44 | 1.39 |
| LABC (11) | 10.14 | 0.35 | 1.86 |
| IBC (10) | 12.76 | 0.48 | 1.22 |
| HER2^+^ (14) | 17.22 | 0.39 | 0.95 |
| LABC (5) | 13.69 | 0.23 | 0.82 |
| IBC (9) | 18.01 | 0.49 | 1.39 |
| M1 (78) |  |  |  |
| HER2^-^ (27) | 14.73 | 0.32 | 1.37 |
| MNIBC (10) | 10.37 | 0.27 | 0.96 |
| MIBC (17) | 16.53 | 0.41 | 1.79 |
| HER2^+^ (51) | 16.08 | 0.59 | 1.35 |
| MNIBC (24) | 16.73 | 0.67 | 1.22 |
| MIBC (27) | 16.08 | 0.53 | 1.66 |

LABC: locally advanced breast cancer; IBC: inflammatory breast cancer; MNIBC: metastatic non-IBC; MIBC: metastatic IBC.
